# Safety of antimalarial medications for use while scuba diving in malaria Endemic Regions

**DOI:** 10.1186/s40794-016-0041-x

**Published:** 2016-10-11

**Authors:** Kyle Petersen, David P. Regis

**Affiliations:** 1 Department of Medicine, Uniformed Services University of the Health Sciences, F. Edward Hébert School of Medicine, 4301 Jones Bridge Rd, Bethesda, MD 20814 USA; 2 Department of Preventive Medicine and Biostatistics, Uniformed Services University of the Health Sciences, F. Edward Hébert School of Medicine, 4301 Jones Bridge Rd, Bethesda, MD 20814 USA

**Keywords:** SCUBA diving, Diving, Malaria, Prophylaxis, Chemoprophylaxis

## Abstract

**Background:**

Recreational diving occurs annually in areas of the world where malaria is endemic. The safety and efficacy of antimalarials for travelers in a hyperbaric environment is unknown. Of particular concern would be medications with adverse effects that could either mimic diving related illnesses such as barotrauma, decompression sickness (DCS) and gas toxicities, or increase the risk for such illnesses.

**Methods:**

We conducted a review of PubMed and Cochrane databases to determine rates of neurologic adverse effects or other effects from antimalarials that may be a problem in the diving environment.

**Results:**

One case report was found on diving and mefloquine. Multiple case reports and clinical trials were found describing neurologic adverse effects of the major chemoprophylactic medications atovaquone/proguanil, chloroquine, doxycycline, mefloquine, and primaquine.

**Conclusions:**

Of the available literature, atovaquone/proguanil and doxycycline are most likely the safest agents and should be preferred; atovaquone/proguanil is superior due to reduced rates of sunburn in the marine environment. Primaquine also appears to be safe, but has reduced efficacy against *P. falciparum*; mefloquine possesses the highest rate of neurologic side effects and therefore these agents should be limited to extreme cases of patients intolerant to other agents. Chloroquine appears unsafe in the hyperbaric environment and should be avoided. More studies are required to include database reviews of returned divers traveling to malaria endemic areas and randomized controlled trials in the hyperbaric environments.

## Background

Recreational Diving with a self-contained underwater breathing apparatus (SCUBA) is a popular hobby. Authors cite there are between 1 and 3 million [1, 2] to 5–9 million [3, 4] persons in the United States and another 90 thousand [2] to 1 million [1] persons in the UK or Europe, respectively, participating actively in Scuba Diving. While figures are hard to come by, one trade source estimated that the sport diving industry generated approximately $3.5 billion in income for equipment suppliers and retailers, course instructors and certification agencies and travel and tourism operators worldwide in 1994 [5]. Due to the large number of participants and the high value to the travel and tourism industry, travel medicine professionals need to be cognizant of this constituency and how to optimally manage their pre-travel care, particularly if they are traveling to malaria endemic regions.

### Hazards inherent to sport diving

“Diving is not dangerous until you forget that it can be” [6]. This quote from the memoirs of a retired North Sea diver sum up the risks of diving nicely; for while the underwater hyperbaric environment poses risks to the diver such as barotrauma, decompression sickness (DCS), hypothermia, drowning and marine animal envenomation, as long as one maintains attention to detail and continuously monitors one’s equipment and environment, the risk of injury is generally very low. The Divers Alert Network reports approximately 90 fatalities a year [4], mostly from drowning [1], and 900–1000 persons requiring hyperbaric treatment for decompression illness [4]. A recent workshop concluded the death rate to be about 0.7 per 100,000 dives and an individual risk per annum of 1in 6000 which was similar to motor vehicle operation or jogging [7]. Unlike driving a motor vehicle or jogging however, the diver has to contend not only with the external environment but also the internal effects arising from the physiologic changes related to pressure exposure. Some of these effects are well known and can be managed, such as direct insult to air filled cavities via barotrauma, DCS from the build-up of inert gas in the tissues and the direct narcotic effects of nitrogen on the central nervous system at depth. However, little is known about how the concomitant use of medications affect the diver while under pressure either directly via changes in cognition for instance or indirectly by affecting their risk for DCS or nitrogen narcosis. Furthermore, as more technologically advanced equipment and gas mixtures (with their own potentially toxic effects) become available to the recreational diver, the margin for error decreases and thus the potential risk for adverse outcomes increases when medication is added to the mix. For instance, oxygen is often used at higher concentrations than that in compressed air to increase time at depth, but comes with the risk of depth-dependent toxicity on both the lungs and more acutely, the risk of central neurologic toxicity, including seizure. Thus a drug that affects seizure threshold may increase the risk of such at depth.

Because of this, many diving physicians will not clear a person for participation in SCUBA diving if they are taking medications with neurocognitive side effects. Furthermore, divers who receive these medications are usually told not to continue diving while taking them. Generally, taking medications while diving is discouraged but not necessarily prohibited due to the lack of clinical studies on safety in the hyperbaric environment, and considerations of the risk/benefit associated with the underlying disease that is being managed. Interestingly, a cross-sectional survey of medication usage in Australian and US divers shed some light on this issue. The authors reported 3–10 % of experienced divers routinely used decongestants, anti-emetics or bronchodilators within 2 h prior to diving, furthermore 25 % of divers were on chronic medications despite no safety data to endorse these practices [8].

In his pioneering work on diving physiology in the 1960s, Bennett studied the effect of several drugs on DCS [9] in rats. Carbachol (cholinergic agonist), glutethimide (an anticonvulsant), phenacetin (an analgesic), epinephrine, scopolamine, pentylenetetrazol, and bemegride (stimulants), all increased incidence of death or spinal cord DCS in these animals. Unfortunately, there are very few human studies on the safety of some medications in diving. Dimenhydrinate, an antihistamine with known sedating side effects induced reduced cognitive capabilities as tested drawing a trail between numbered circles at 10 and 30 meters of sea water (msw) in a hyperbaric chamber, but 5 other cognition tests showed no impairment [10]. On the other hand, both clemastine, another CNS active antihistamine, and pseudoephedrine had no effect on subjects in a hyperbaric environment, demonstrating that some medications are indeed safe to use [11, 12].

In addition to the neurologic side effects, SCUBA diving also alters cardiovascular physiology due to immersion and relative hypothermia, both of which cause central shunting and peripheral vasoconstriction that would alter volume of distribution and consequently renal clearance and hepatic redistribution rates [13, 14]. Depending on pharmacokinetics, these changes could therefore affect both the efficacy and side effect profile of a medication, and reciprocally a drug could alter these physiologic responses by changing tissue gas dynamics and thus risks for DCS and toxic effects of pressurized breathing gases. Because diving related illnesses require management by a specialist and often recompression in a chamber, both of which may be hours away by automobile or aircraft, diagnostic decisions confounded by medications could delay therapy or unnecessarily utilize resources, putting the patient at undue risk of a medevac if an incorrect diagnosis of a diving illness is made.

### Risks of malaria to the diver

Due to great underwater visibility, temperature and the colorful variety of marine life encountered, tropical destinations are popular and among the most highly visited by divers [2]. Invariably, these destinations will include countries where malaria is endemic, indeed a 2012 trade publication cited countries such as Honduras, Indonesia, the Philippines, Malaysia, Thailand as among the “top 100 readers choice” places to go diving. Diving vacations are usually conducted from either shore based resorts which has shore diving or transports the divers out to the dive site in a boat. The majority of these shallow dive sites therefore are close to land and potentially place the diver at risk of malaria acquisition if they are diving in an endemic area. Alternatively, there are “live-aboard” vessels constructed or modified specifically to house diving operations during the day and berth the divers at night. Live aboard boats should not be assumed to be safe from malaria transmission however. Radio labeled *Anopheles* spp. mosquitoes have been shown to fly 2 miles in studies [15] suggesting that any diver on shore or on board a boat is at risk for malaria and therefore should be given counseling on prevention if diving in a country where malaria is endemic.

## Review of antimalarial agents

The most effective protective measure against malaria is the use of oral prophylactic anti-malarial drugs taken prior to exposure. This is particularly true where topical preventative agents like DEET and permethrin-impregnated clothing are not an option due to the periodic immersion in seawater. The safety of oral antimalarials in diving however is unknown and they have not been evaluated in humans or animals [16]. Given the concerns about altered physiology and side effects of medications in a hyperbaric environment, a review of the available literature is desperately needed. A review of PUBMED articles using the keywords “diving” and “malaria” resulted in only 1 article, a letter to the editor advocating the avoidance of mefloquine and adoption of doxycycline as the preferred antimalarial in diving [17, 18]. Indeed, a table from another publication indicates an absence of existing studies [16]. We will therefore review the side effects of various antimalarials with a focus on ones that could possibly mimic symptoms of barotrauma, DCS or gas toxicity; or alternatively, affect the diver’s risk thereof. A search was conducted of PUBMED and Cochrane databases using the keywords atovaquone, proguanil, chloroquine, primaquine, mefloquine, doxycycline, chemoprophylaxis, malaria, adverse effect, side effect, neurologic, psychiatric, neurocognitive and central nervous system.

### Chloroquine

Chloroquine has long been used for both the prevention and treatment of malaria. Unfortunately, due to widespread use for over three quarters of a century, its effectiveness as a malaria prophylactic is limited to just a few areas of the world, many of which are popular diving destinations. It is, however, still used worldwide in conjunction with proguanil for prophylaxis in chloroquine resistant areas as a cost effective alternative to some of the more expensive antimalarial medications, demonstrating a 72 % (95 % CI: 56 to 82 %) effectiveness in a 1993 study with similar findings in more recent studies [19–21]. Furthermore, it is used for diseases other than malaria, including the management of rheumatologic disorders and treatment of amebiasis [22, 23]. Conversely, hydroxychloroquine sulfate, a drug primarily used for treatment of rheumatologic conditions can be used in place of chloroquine [24]. For the prevention of malaria, chloroquine is generally initiated with or without proguanil 1–2 weeks prior to travel and continued for 4 weeks upon return [24]. It is taken once weekly, making it convenient for long term use. Because it is one of the older antimalarials, its side effect profile is one of the better known and studied.

The more commonly reported side effects include gastrointestinal disturbance, headache, dizziness, blurred vision, insomnia, and pruritus; all are generally mild and normally do not require discontinuation of the drug [19, 24]. In a multicenter, randomized, double blind study of non-immune travelers to sub-Saharan Africa, the chloroquine-proguanil (C-P) arm had the highest proportion of mild to moderate adverse events (69/153; 45 %, 95 % CI: 37 to 53 %) but only 8 withdrawals from the group of 153 (5 %, 95 % CI: 2 to 9 %). This was not statistically different (*p* = 0.425) from the other treatment arms which included atovaquone-proguanil (A-P) (*n* = 3; 2 %, 95 % CI: 0 to 4 %), mefloquine (*n* = 6; 4 %, 95 % CI: 1 to 8 %) and doxycycline (*n* = 5; 3 %, 95 % CI: 0 to 6 %) [19]. These numbers were similar to earlier studies [21] and although manageable, as demonstrated by the low withdrawal rate, these symptoms could be confused with DCS. Also, while many of the symptoms may manifest during the pre-travel/diving period and thus be dealt with accordingly, others may not present until diving has commenced. Furthermore, besides being DCS confounders they may certainly affect a diver’s performance and could lead to a diving mishap if they are not managed appropriately.

Though rare but of legitimate concern, are the much more severe neuro-psychiatric side effects attributed to chloroquine. Severe symptoms of headache, strange or vivid dreams, dizziness, anxiety, depression, sleeplessness, and visual disturbance were noted to be 4 % (95 % CI: 1–7 %) in one study, while a population-based observational study using a database of 35,370 patients showed an incidence rate for depression, psychosis and panic attacks to be 7.6 % (95 % CI 5.5–10.5), 0.4 (95 % CI 0.1–1.6) and 1.3 % (95 % CI 0.6–2.8), respectively [19, 25]. Furthermore, numerous cases of seizures have been reported in patients receiving chloroquine in varying doses for both prophylaxis and treatment of malaria and other disorders with good demonstration of cause and effect [21, 26–28]. The mechanism is thought to be related to the inhibition of GABAergic neurotransmission [28]. This is particularly concerning as there is some evidence that this pathway is involved in the mechanism by which the neuroleptic vigabatrin prevents the seizures associated with hyperbaric oxygen toxicity. This suggests that chloroquine could possibly lower the seizure threshold in divers, particularly those diving oxygen enriched mixtures such as Nitrox [29]. It is because of this seizure risk that many militaries avoid prescribing this medication to its divers deploying to malaria endemic areas.

While its low cost and relatively tolerable side effect profile make it an attractive option for the general population, the myriad of mild to severe adverse effects that both mimic and possibly potentiate the spectrum of illnesses that could afflict a diver prohibit its prescription in this population.

### Mefloquine

Mefloquine is a readily available medication worldwide and is available as a generic making it a cost effective choice for prophylaxis; it is generally started 2 weeks prior to travel. Mefloquine’s once weekly dosing schedule make it a convenient agent for those on long term trips to the tropics, such as expatriates and military forces on deployment where adherence can be an issue. In addition, it is not photosensitizing (an advantage to patients in a mostly marine or beach environment) and does not cause gastric irritation. However, the drug has well established central nervous system side effects estimated between 1/607 and 20,000 patients [16], manifestations that could be mistaken for DCS or arterial-gas-embolism-related neurological symptoms in a diver [17]. This could result in subsequent inappropriate hyperbaric treatment and or aeromedical evacuation, hence the author’s recommendation against its use in sport divers at risk for malaria acquisition [18]. Initial studies in over 600 travelers to Africa quoted a 10 % adverse neuropsychiatric side effect rate, with 17 % described as moderate and 4 % as severe. A recent study of more than 1,000 Peace Corps volunteers taking malaria prophylaxis over a 1-year period showed 65 % had some neuropsychiatric side effect (23 % mild, 31 % moderate and 10 % severe) requiring 35 % to seek medical care, 1 % to be hospitalized and 28 % to change prophylaxis regimens [30]. However, most diving trips are not for prolonged periods of time and the relatively unique isolation experienced by Peace Corps volunteers immersed in a foreign culture may introduce psychosocial stressor bias inapplicable to dive travel. In a head-to-head trial against chloroquine, mefloquine caused significantly higher rates of vivid dreams, depression, dizziness, nausea, irritability and tremor. Neuropsychiatric adverse events occurred between 5 and 10 % of subjects [31]. In a trial comparing mefloquine to doxycycline, there were higher rates of headache, vertigo and neuropsychiatric events in the mefloquine arm [32]. Finally, in comparison with atovaquone/proguanil, a study of 1013 patients in 15 travel clinics showed the rate of all described neuropsychiatric side effects was twice as common (29 % vs 14 %) in patients using mefloquine prophylaxis, and 19 (4 %) of patients had to discontinue therapy in the mefloquine arm versus only 3 (0.6 %) in the atovaquone/proguanil arm [33]. However, in tests of complex flying or driving performance using simulators, there was no significant psychomotor impairment even when coupled with ethanol, suggesting that side effects may be milder and more tolerable than reported and the drug may have a safe profile for diving [34, 35]. Unfortunately, Mefloquine has a United States Food and Drug Administration, Australian Therapeutic Goods Administration and United Kingdom Medicines and Healthcare products Regulatory Agency “black box” (or country equivalent) warning included in the package inserts stating that the risk of vestibular and neuropsychiatric adverse reactions may persist indefinitely and all patients must receive a medication guide and wallet card with their prescription. This warning and potential risk for litigation has diminished provider enthusiasm to prescribe this medication in the United States.

In summary, Mefloquine has the highest rates of CNS side effects of all prophylactic anti-malarial medications occurring in 10 to 30 % of patients and thus should be a last option for diving vacations where prophylaxis is required due to its potential to mimic dysbaric symptoms. However, certain patients preparing for dive travel to a malarious area may be a candidate for use if they are intolerant of all other anti-malarial medications. Especially if there is the opportunity for a trial dose and observation for side effects prior to travel, with strict instructions to discontinue the medication at first sign of adverse events.

### Doxycycline

Doxycycline is has been in clinical use for almost half a century for both its antimicrobial properties as well as its anti-inflammatory ones. Its uses include the management of soft tissue infections, atypical bacterial infections and malaria, as well as acne vulgaris and rosacea where sub-therapeutic doses are thought to control the inflammatory component of the disease [36–38] although all these indications require a higher dose than malaria prophylaxis. Like chloroquine, its low cost makes it an attractive anti-malarial prophylactic and because of its proven effectiveness as a prophylactic, it is listed as one of the accepted alternatives for such [36]. In addition, it is convenient for last minute travelers as it is started only a few days before travel, and may already be prescribed in the same doses for acne. It would also prevent other infections that an adventurous traveler, like a diver, may come in contact with, including leptospirosis and rickettsial infections. Disadvantages are its requirement for daily dosing as well as its toxicities in certain populations such as pregnant women and children under 8 years old. Furthermore, its side effect profile, though not generally prohibitive to its use (even in divers), requires proper counseling to the traveler to assure both proper measures are taken to mitigate their likelihood as well as self-monitoring for their onset to ensure prompt and appropriate management.

The list of all possible side effects for doxycycline is quite long and the reader is directed elsewhere to the numerous drug reference sources available for a comprehensive list. The focus here will be on those that could be confused with DCS or place the diver at risk for such, ranging from the common related to gastrointestinal tract and skin to the rarer and more idiosyncratic which affect the central nervous system. Nausea (13 %) and vomiting (8 %) are among the more commonly reported side effects and while protean, can certainly be a presenting sign of the more constitutional DCS symptoms [39]. Fortunately these can be mitigated by avoiding administration on an empty stomach and assuring adequate hydration [39]. The latter precaution is also important because the tablets should be taken with plenty of water and not before bedtime, i.e. well prior to laying down, to avoid the pill esophagitis commonly associated with doxycycline and whose symptoms could be mistaken for a diving induced pneumomediastinum, particularly if there is either an esophageal ulcer or perforation present [38]. It should be further noted that there are different formulations of doxycycline and while there have been no direct comparisons of the different forms in clinical trials there is some evidence that the hyclate salt form causes more gastrointestinal side effects than the monohydrate or enteric coated hyclate formulation presumably due to its higher acidity [40]. Thus, selected prescribing of particular salt formulations along with proper patient education on administration could have a considerable effect on lowering the incidence of gastrointestinal side effects.

Photosensitivity is another commonly associated effect of doxycycline administration with rates between 3 and 10.7 % and manifestations ranging from mild papular erythema to exfoliative dermatitides [39, 41, 42]. This has been shown to be dependent both on the dose as well as the intensity of sunlight exposure [41, 43]. This is a particular problem for recreational divers in that cutaneous DCS can present similarly and diving often occurs in tropical environments where there is constant exposure to heat and bright sunlight, posing the dilemma of staying covered versus the risk of overheating and dehydration. While liberal and frequent use of appropriate sunblock can mitigate this effect it is no guarantee with severe dermatitis having been reported even with adequate sunblock use [42]. The one helpful delineator between cutaneous DCS and doxycycline phototoxicity is the predilection of the latter for sun-exposed areas and the former for a truncal distribution. Finally, doxycycline has neurological side effects ranging from the more common headache (2 %) and dizziness to the rarer but concerning intracranial hypertension and its associated symptoms, which all can mimic the more severe neurological presentation of DCS [38, 39]. While tetracycline is more commonly implicated with the latter side effect it has been described with doxycycline to include those taking it for malaria prophylaxis [44–47]. Fortunately, the incidence for all idiopathic intracranial hypertension is rare with estimates in the United States being 0.9 per 100,000 persons/year, making this an uncommon but noteworthy side effect in the diver for whom doxycycline is being considered [48]. A study of 22 rabbits under hyperbaric conditions demonstrated a 76 % increase in Blood Brain Barrier permeability and higher CSF levels for tetracycline than controls, clearly showing that diving alone affects pharmacology. There was no statistical difference in DCS rates (7 DCS vs. 9 No DCS) among rabbits with elevated brain antibiotic levels, but this is a very underpowered study to comment on the safety of doxycycline in diving and it did not address oxygen toxicity [49].

In summary, while doxycycline is a recommended second line agent for malaria prophylaxis due to its cost, effectiveness and short period of pre-travel dosing, its side effect profile, though manageable, keeps it in the alternate category for the traveling diver as well.

### Atovaquone/proguanil

Atovaquone/proguanil (A-P) is available in many countries as a co-formulated tablet for both adult and pediatric patients. It appears to be very well tolerated with minimal side effects, however it must be taken daily starting 2 days prior to travel and continued on a daily basis throughout the trip and for a week afterwards; it is also quite costly. In a double blind placebo controlled efficacy and toxicity study of more than 1000 patients conducted in 21 travel clinics in Europe, Canada and South Africa, A-P was compared to chloroquine plus proguanil and adverse events were monitored at day 7 and 28 after leaving a malarious region [21]. Twenty-two percent of participants reported adverse effects from A-P with half being gastrointestinal and the other half (10 %) neuropsychiatric; the majority of these side effects were mild. Strange or vivid dreams were most common in among neuropsychiatric side effects (4 %), followed by dizziness (3 %) and visual difficulties (2 %), but no neuropsychiatric side effect appeared more common than chloroquine/proguanil with statistical significance and none required stopping the medication. Only 11 persons (10 in the C-P and one in the entire A-P arm) discontinued medication due to drug attributable side effects (A-P drug allergy not neuropsychiatric).

A-P was also evaluated in a randomized double blind, placebo controlled trial compared to mefloquine among 1000 patients presenting to travel clinics in Europe and South Africa [33]. Patients received mefloquine placebo followed by active A-P or active mefloquine followed by A-P placebo; the mefloquine was started 3 weeks before travel and the A-P 2 days before and continued for 30 or 7 days after return, respectively. Thirty percent of patients in the A-P arm had drug attributable side effects, and of those 14 % were neuropsychiatric, with the majority being strange dreams (7 %), insomnia (3 %) and dizziness (2 %). Adverse effects were significantly less common (2–4 fold) than mefloquine however, and only 3 of 493 persons in the entire trial receiving A-P had to discontinue drug due to adverse effects. The investigators believed the higher rate of neuropsychiatric side effects observed compared to the previous trial against C-P was attributable to mefloquine placebo effect as symptoms often appeared prior to active A-P use.

A third trial conducted in a travel clinic in the Netherlands again randomized 140 patients to mefloquine placebo plus A-P or A-P placebo plus mefloquine [50]. One hundred nineteen patients completed the study and all patients were screened repeatedly with 2 mood index tools. There were no changes noted in tension, depression, anger, fatigue or vigor from baseline in patients taking A-P. This was a marked contrast to patients in the mefloquine arm who experienced significant deterioration in depression, fatigue, anger and vigor. Concentration skills such as hand eye coordination or coding speed were not affected by either medication.

Finally, a Canadian study of A-P vs. primaquine in 28 pilots revealed no significant impact on neurocognitive testing like serial reaction, subtraction, or logical reasoning and no impact on flight simulator performance [51]. There was also no increase in physical fatigue, sleepiness, or mental fatigue. Overall, A-P has one of the lowest neuropsychiatric side effect profiles ranging between 10 and 14 % in 2 large trials, and has other side effects that are generally gastrointestinal in nature, although most patients report all side effects as mild and none required discontinuation for neurological intolerability. The rate of side effects may be overstated in these initial trials however, due to mefloquine placebo bias and drug approval symptom recording methods as no adverse effects were detected in subsequent intensive mood index and neurocognitive trials. Because of its relatively low side effect profile, especially those that could mimic or potentiate a diving related illness, A-P is considered a preferred option for diving travel to malarious regions ahead of all other agents.

### Primaquine

Primaquine is an 8-aminoquinoline anti-malarial developed for treatment of *P. vivax* or *P. ovale* malaria to prevent relapse. It is now being studied as a gametocytocidal agent in *P. falciparum* eradication efforts. In addition, it has been studied as primary prophylaxis against both *P. falciparum* and *P. vivax* malaria where it has showed an 85–93 % protective efficacy [52, 53]. These studies resulted in Primaquine’s inclusion as an recommended alternative agent for malaria chemoprophylaxis [24], however it is not approved for this use by regulatory agencies and must be used “off-label” The dosing however, has the highest pill burden of all prophylaxis at 2 pills a day started 2 days before travel and continued for a week after returning. In a Canadian study of primaquine vs A-P, neurocognitive impact in 28 pilots revealed no significant impact on neurocognitive testing like serial reaction, subtraction, or logical reasoning and no impact on flight simulator performance. There was also no incidence of sleepiness, mental fatigue, or physical fatigue [51]. Observed side effects included abdominal cramps, headache, nausea and diarrhea. All symptoms predominated in the first week, often improved subsequently, and were mild in nature. Other sources suggest GI side effects are mild and can be minimized or eliminated by taking the medication with food [24]. The sole incidence of CNS side effects from primaquine described in the literature is a case report of a 55 y/o male who developed depression, anorexia, confusion, auditory hallucinations, 2 days after starting 15 mg of primaquine; the symptoms resolved 24 h after discontinuing medication [54]. Overall, primaquine’s low side effect profile and the relative lack of CNS side effects make it an attractive option for diving travel, however its relatively lower protective efficacy against malaria and higher pill burden relegate it to a second line option compared to other agents.

## Conclusions

Table 1 contains a summary of recommended prophylaxis for dive related travel to malaria infected regions; it should be noted that the level of evidence is low around CII for most chemoprophylactic agents. Based on available evidence and knowledge of diving physiology, it is our opinion the preferred regimen to prevent malaria in this group of travelers is atovaquone-proguanil due to its low side effects profile and high protective efficacy against P falciparum malaria. Doxycycline as an acceptable alternative, assuming proper counseling and precautions, and would be equivalent except for its rare side effect of intracranial hypertension and its propensity to photosensitization, especially in the marine environment. Furthermore, because atovaquone-proguanil and doxycycline are both currently used by the U.S. Navy aviation community and are considered to not affect cognitive function in pilots, they are logical candidate drugs for diving operations as well. However, the daily dosing schedule can decrease adherence, possibly causing breakthrough cases of malaria. In patients intolerant or allergic to these 2 agents, Primaquine is a good alternative due to its lack of neurocognitive side effects, but it has a higher pill burden and lower efficacy than A-P or doxycycline against falciparum malaria. Mefloquine is the drug of last resort due to its impact on sleep, mood and neurologic side effects, however since it does not appear to impact psychomotor function and many patients do not get side effects it could be used in the rare patient intolerant to all other agents, provided test doses are given and the patient observed for side effects prior to travel. Chloroquine should not be used due to its risk to induce seizures. For all chemoprophylactic agents, safety in relation to oxygen toxicity or DCS remains a huge unanswered question with limited studies available for review. Clearly the paucity of data in this realm calls for more research.

**Table 1:** Antimalarial Prophylaxis Recommendations for Dive Related Travel

Prophylactic medication	Recommendation	Comments
Atovaquone-proguanil 250/100 mg daily	Primary regimen	• Lowest and most benign side effect profile
Doxycycline 100 mg daily	Secondary regimen	• Phototoxicity and esophageal effects make second line
		• Very rare but real risk of reversible intracranial hypertension
Primaquine phosphate 52.6 mg daily	Alternative regimen	• Lower level of protective efficacy than other agents
		• High pill burden
Mefloquine 250 mg weekly	Alternative regimen	• Prophylaxis of last resort
		• High levels of CNS side effects may mimic DCS
		• Possibility with test doses prior to travel or in treatment experienced patient
Chloroquine phosphate 500 mg weekly	Not recommended	• Geographically limited efficacy alone; still relatively low with proguanil
		• Known association with seizures
		• Theoretical possibility of eliciting hyperbaric oxygen seizure
		• Other CNS side effects

## Abbreviations

A-P: Atovaquone/proguanil
CNS: Central nervous system
DCS: Decompression Sickness
SCUBA: Self-contained underwater breathing apparatus.
